# Draft genome sequence of *Actinomadura* sp. K4S16 and elucidation of the nonthmicin biosynthetic pathway

**DOI:** 10.7150/jgen.44650

**Published:** 2020-05-17

**Authors:** Hisayuki Komaki, Enjuro Harunari, Natsuko Ichikawa, Akira Hosoyama, Moriyuki Hamada, Kannika Duangmal, Arinthip Thamchaipenet, Yasuhiro Igarashi

**Affiliations:** 1 Biological Resource Center, National Institute of Technology and Evaluation (NBRC), Kisarazu, Chiba 292-0818, Japan.; 2 Biotechnology Research Center and Department of Biotechnology, Toyama Prefectural University, Imizu, Toyama 939-0398, Japan.; 3 NBRC, Shibuya-ku, Tokyo 151-0066, Japan.; 4 Faculty of Science, Kasetsart University, Bangkok, Thailand.

**Keywords:** *Actinomadura*, biosynthesis, nonthmicin, polyether, polyketide, tetronate

## Abstract

*Actinomadura* sp. K4S16 (=NBRC 110471) is a producer of a novel tetronate polyether compound nonthmicin. Here, we report the draft genome sequence of this strain together with features of the organism and assembly, annotation and analysis of the genome sequence. The 9.6 Mb genome of *Actinomadura* sp. K4S16 encoded 9,004 putative ORFs, of which 7,701 were assigned with COG categories. The genome contained four type-I polyketide synthase (PKS) gene clusters, two type-II PKS gene clusters, and three nonribosomal peptide synthetase (NRPS) gene clusters. Among the type-I PKS gene (*t1pks*) clusters, a large *t1pks* cluster was annotated to be responsible for nonthmicin synthesis based on bioinformatic analyses. We also performed feeding experiments using labeled precursors and propose the biosynthetic pathway of nonthmicin.

## Introduction

**Figure 1:**
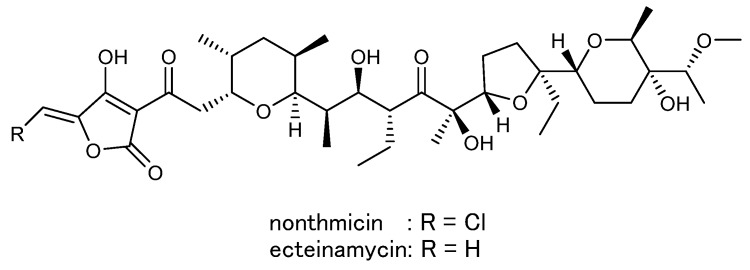
Chemical structures of nonthmicin and ecteinamycin.

Actinomycetes are well known as a promising source for diverse bioactive secondary metabolites. Especially, members of *Streptomyces* have attracted attention as the most useful screening sources for new drug leads and a large number of bioactive compounds have been identified from cultures of this genus 1,2. Consequently, the chance of finding novel secondary metabolites from *Streptomyces* members has recently dwindled. Thus, the focus of screening has recently moved to less exploited genera of rare actinomycetes 3. In our screening for novel bioactive compounds from rare actinomycetes, *Actinomadura* sp. K4S16 was isolated from rice field soil in Thailand and found to produce a tetronate polyether designated nonthmicin along with ecteinamycin (Fig. 1) 4. Nonthmicin shows inhibitory activity against tumor cell invasion and protective activity for neuronal cell damage. This new polyether compound is characterized by the tetronic acid functionality modified by a chlorine atom. Halogenated tetronic acids are not known from nature except nonthmicin. In this study, we conducted whole genome shotgun sequencing of the strain to elucidate the biosynthetic pathway of nonthmicin. We herein present the draft genome sequence of *Actinomadura* sp. K4S16, together with the taxonomical identification of the strain, description of its genome properties and annotation of the gene cluster for nonthmicin biosynthesis. Biosynthetic pathway for nonthmicin was predicted by bioinformatics analysis and confirmed by precursor-incorporation experiments.

## Materials and Methods

### Sequenced strain

In the course of screening for novel bioactive substances from rare actinomycetes, *Actinomadura* sp. K4S16 was isolated from rice field soil collected in Thailand and found to produce a novel polyketide compound named nonthmicin and its known congener ecteinamycin (Fig. 1) 4. *Actinomadura* sp. K4S16 was preserved as TP-A0891 at the Toyama Prefectural University, deposited into the NBRC culture collection, and publicly available from the collection as NBRC 110471.

### Chemotaxonomic analyses

The isomer of diaminopimelic acid in the whole-cell hydrolysate was analyzed according to the method described by Hasegawa *et al*. 5. Isoprenoid quinones and cellular fatty acids were analyzed as described previously 6.

### Phylogenetic analysis based on 16S rRNA gene sequences

PCR template was prepared according to the protocol for Gram-positive bacteria of DNeasy Blood & Tissue kit (Qiagen). The gene encoding 16S rRNA was amplified by PCR using two universal primers, 9F and 1541R. After purification of the PCR product by AMPure (Beckman Coulter), the sequencing was carried out according to an established method 7. Homology search of the sequence was conducted using EzBioCloud 8. A phylogenetic tree was reconstructed by on the basis of the 16S rRNA gene sequence together with taxonomically close type strains showing more than 98% similarities by ClustalX2 9.

### Growth conditions and genomic DNA preparation

A monoisolate of* Actinomadura* sp. K4S16, isolated as single colony, was grown on polycarbonate membrane filter (Advantec) on double-diluted NBRC 227 agar medium (0.2% yeast extract, 0.5% malt extract, 0.2% glucose, 2% agar, pH 7.3) at 28°C. High quality genomic DNA for sequencing was extracted and isolated from the mycelia with an EZ1 DNA Tissue Kit and a BioRobot EZ1 (Qiagen) according to the manufacturer's protocol for extraction of nucleic acid from Gram-positive bacteria. The size, purity, and double-strand DNA concentration of the genomic DNA were measured by pulsed-field gel electrophoresis, ratio of absorbance values at 260 nm and 280 nm, and Quant-iT PicoGreen dsDNA Assay Kit (Life Technologies), respectively, to assess the quality of genomic DNA.

### Genome sequencing and assembly

Shotgun and paired-end libraries were prepared and subsequently sequenced using 454 pyrosequencing technology and MiSeq (Illumina) paired-end technology, respectively (Table 1). The 82 Mb shotgun sequences and 707 Mb paired-end sequences were assembled using Newbler v2.8 and subsequently finished using GenoFinisher 10 to yield 43 scaffolds larger than 500 bp. The draft genome sequence has been deposited in the INSDC database under the accession number BDDE01000001-BDDE01000043. The project information and its association with MIGS version 2.0 compliance are summarized in Table 1 11.

### Genome annotation

Coding sequences were predicted with Prodigal 12 and tRNA-scanSE 13. The gene functions were assigned using an in-house genome annotation pipeline, and domains related to polyketide synthase (PKS) and nonribosomal peptide synthetase (NRPS) were searched using the SMART and PFAM domain databases. PKS and NRPS gene clusters and their domain organizations were determined as reported previously 7. Substrates of adenylation (A) and acyltransferase (AT) domains were predicted using antiSMASH 14. Protein-protein BLAST search against the NCBI Non-redundant protein sequences (nr) database was also used for predicting function of proteins encoded in the nonthmicin biosynthetic gene cluster.

### Digital DNA-DNA hybridization

Digital DNA-DNA hybridization (DDH) between *Actinomadura* sp. K4S16 and *A. mexicana* DSM 44485^T^ (FZNP01000001-FZNP01000053) was conducted using Formula 2 of Genome-to-Genome Distance Calculator 2.1 15.

### Feeding experiments using labeled precursors

Inoculation, cultivation, extraction, and purification were performed in the same manner as previously reported 4. Supplementation of sodium [1-^13^C]acetate or [1-^13^C]propionate (20 mg/100 ml medium/flask, 10 flasks) was initiated at 48 h after inoculation and periodically carried out every 24 h for four times. After further incubation for 24 h, the whole culture broths were extracted with 1-butanol and several steps of purification yielded 55 mg and 100 mg of ^13^C-labeled nonthmicin, respectively.

## Results and Discussion

### Feature, classification, and genome properties

**Figure 2:**
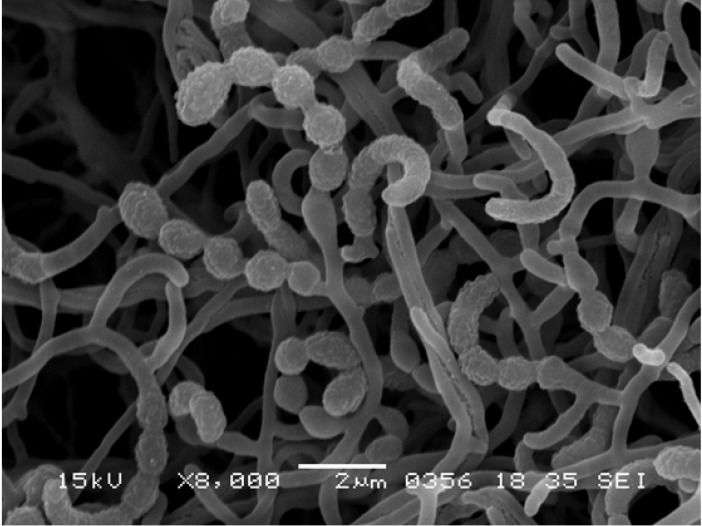
Scanning electron micrograph of *Actinomadura* sp. K4S16 grown on double-diluted ISP 2 agar for 7 days at 28 °C. Bar, 2 µm.

The general feature of *Actinomadura* sp. K4S16 is shown in Table 2. This strain grew well on ISP 2 and ISP 4 agar media, but poorly on ISP 5 and ISP 7. The color of aerial mycelia was white and that of the reverse side was pale orange on ISP 2 agar medium. Strain K4S16 formed extensively branched substrate mycelium. The aerial mycelium formed short chains of arthrospores. A scanning electron micrograph of this strain (Fig. 2) shows that spore chains are hooked or spiral (1 turn) and the spore surface is rugose. Growth occurred at 20-45 °C (optimum 28 °C) and pH 5-8 (optimum pH 7). Strain K4S16 exhibited growth with 0-2 % (w/v) NaCl (optimum 0 % NaCl) and the strain utilized arabinose, fructose, glucose, mannitol, rhamnose, sucrose, and xylose as sole carbon source for energy and growth, but not raffinose (all at 1%, w/v).

**Figure 3:**
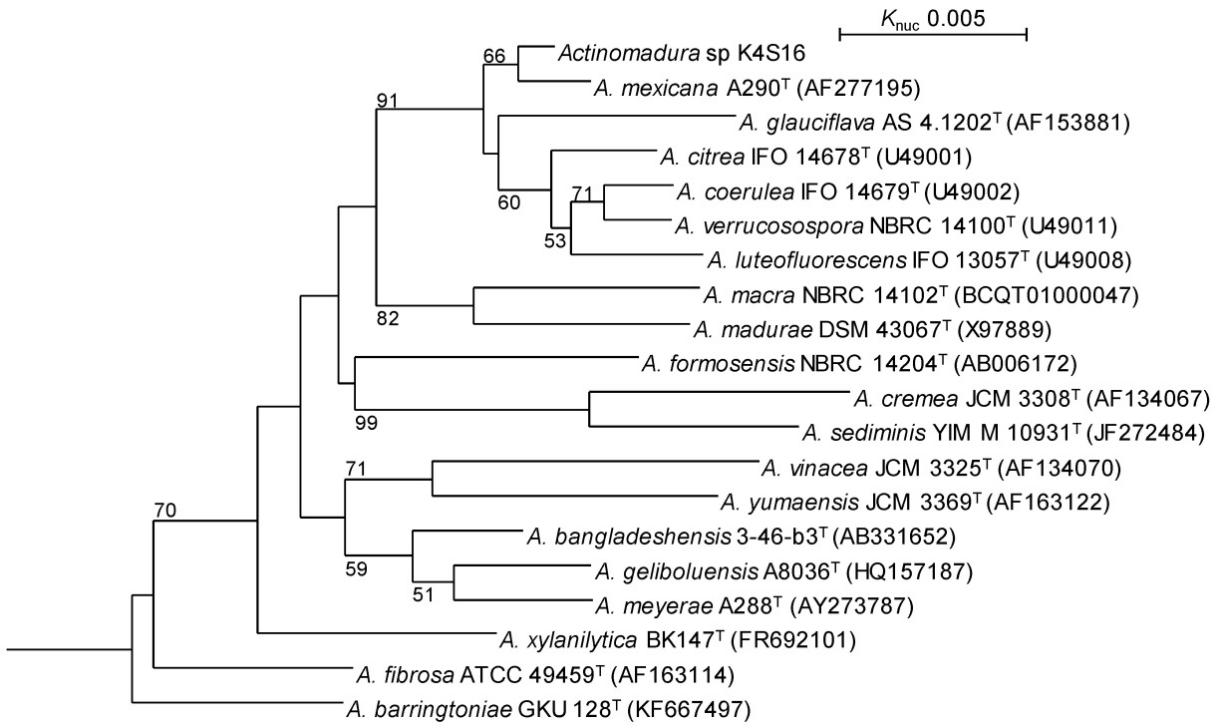
Phylogenetic tree based on 16S rRNA gene sequences of *Actinomadura* sp. K4S16 and its phylogenetically close type strains showing over 98.0 % sequence similarities. Accession numbers for 16S rRNA genes are shown in parentheses. The tree uses sequences aligned by ClustalX2 9 and constructed by the neighbor-joining method 24. All positions containing gaps were eliminated. The building of the tree also involves a bootstrapping process repeated 1,000 times to generate a majority consensus tree, and only bootstrap values above 50% are shown at branching points. *Streptosporangium roseum* DSM 43021^T^ was used as an outgroup.

The whole-cell hydrolysate of strain K4S16 contained *meso*-diaminopimelic acid as its diagnostic peptidoglycan diamino acid. The predominant menaquinones were identified as MK-9(H_4_) and MK-9(H_6_); in addition, MK-9(H_2_) and MK-9(H_8_) were also detected as minor components. The major cellular fatty acids (>10%) were C_16:0_ and C_18:1_ *ω*9c. The 16S rRNA gene sequence of the strain indicated the highest similarity (99.58 %, 1415/1421) to *Actinomadura mexicana* A290^T^ (AF277195) as the closest type strain. The phylogenetic analysis confirmed that the strain belongs to the genus *Actinomadura* (Fig. 3).

A draft genome size of *Actinomadura* sp. K4S16 was 9,647,292 bp and the G+C content was 72.4 % (Table 3). Of the total 9,068 genes, 9,004 were protein-coding genes and 64 were RNA genes. The classification of genes into COGs functional categories is shown in Table 4. Digital DDH between *Actinomadura* sp. K4S16 and the type strain of the closest species, *A. mexicana* DSM 44485^T^ suggested that the DNA-DNA relatedness was 49.0 %, which is below 70 %, the cut-off point for the assignment of bacterial strains to the same species 16. This suggests that *Actinomadura* sp. K4S16 is a novel independent genomospecies.

### PKS and NRPS gene clusters in the genome

We analyzed biosynthetic gene clusters for polyketides and nonribpsomal peptides in the genome. *Actinomadura* sp. K4S16 harbored four type-I PKS gene (*t1pks*) clusters, two type-II PKS gene (*t2pks*) clusters, and three NRPS gene (*nrps*) clusters, as shown in Table 5. *T1pks-1* cluster encoded only a PKS composed of ACP-KS/AT/DH/KR/ACP/ACP-TE domains, which showed 87% sequence identity to phenolpthiocerol synthesis type-I polyketide synthase PpsD of *Mycobacterium tuberculosis* 401416 (CND43678), suggesting it may synthesize phenolpthiocerol-like compounds. *T1pks-2* cluster encoded two PKSs whose domain organizations are KS/AT/KR and KS/AT, respectively. Since these PKSs did not show sequence similarities to PKSs whose products are identified and the domain organization is unusual, we are not able to predict the product. *T1pks-3* cluster encoded a PKS composed of KS/AT/KR/DH domains. Because such domain organization is specific to iterative PKSs for enediyne syntheses, this gene cluster likely synthesizes enediyne-type polyketide compounds. *T1pks-4* cluster is responsible for nonthmicin synthesis as stated in the following section. *T2pks-1* cluster might synthesize aromatic compounds similar to tetarimycin A or mithramycin, because its KSα showed 70 to 71 % sequence identities to TamM (AFY23044) and MtmP (CAA61989). *T2pks-2* cluster did not show high sequence similarities (less than 55 % identities) to any PKSs registered in GenBank, suggesting that the product will be unique. *Nrps-1* gene cluster harbored six NRPS modules and the products were predicted to be peptides containing amino dihydroxybenzoic acid, cysteine, glycine, and methyl ornithine. *Nrps-2* gene cluster encoded four modules and the products will be composed of starter molecule-Cys-Cys-methyl Cys. *Nrps-3* gene cluster had seven modules and the products are likely hexapeptides including amino acid residues such as alanine and threonine. The presence of these PKS and NRPS gene clusters suggests that this strain has the potential to produce diverse polyketide- and nonribosomal peptide-compounds as the secondary metabolites.

### Nonthmicin biosynthetic pathway

**Figure 4:**
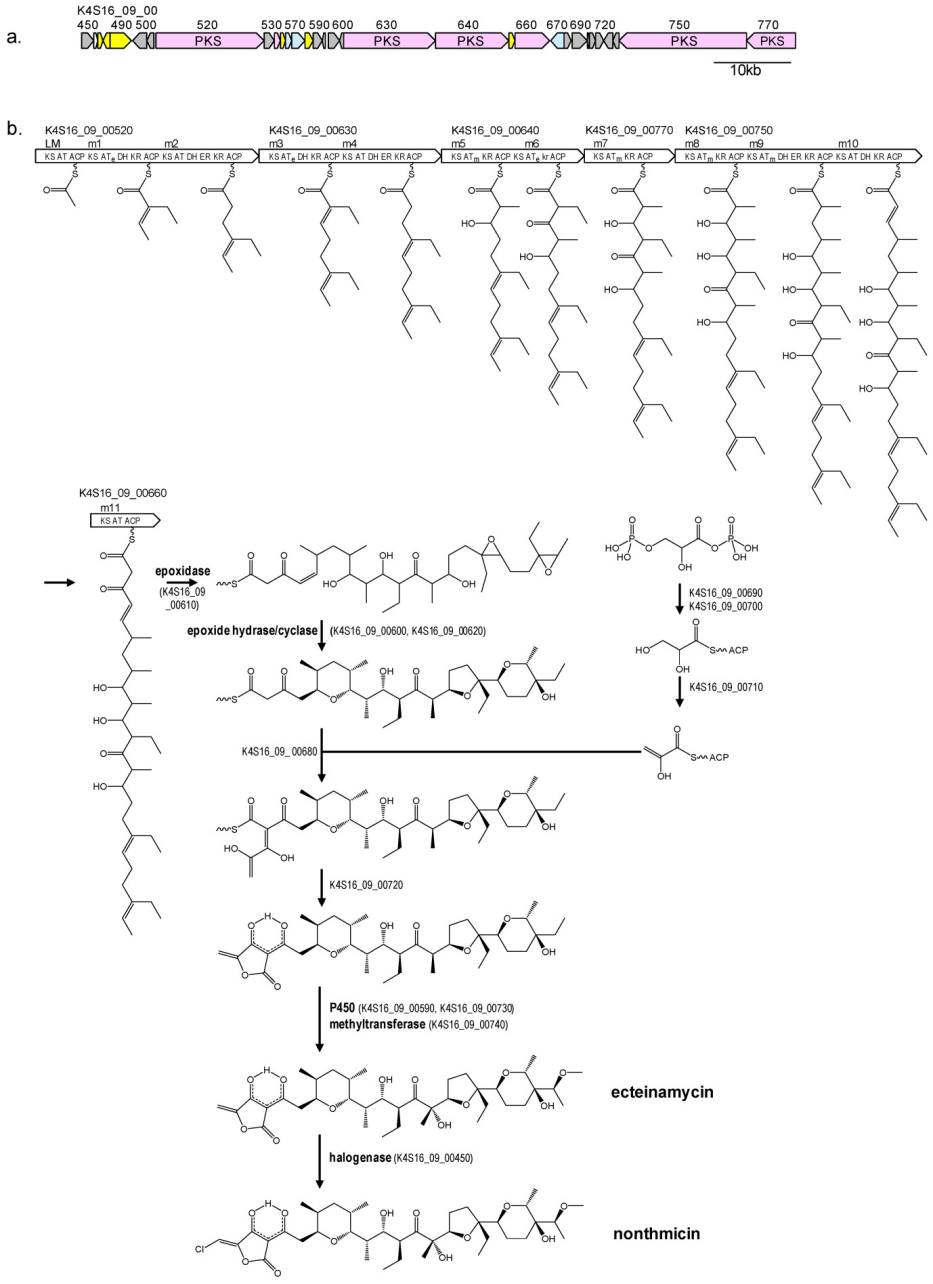
Genetic map of nonthmicin biosynthetic gene cluster of *Actionomadura* sp. K4S16 (a) and the predicted biosynthetic pathway (b). Pink, PKS; yellow, transcriptional regulator; light blue, transporter; gray, genes related to secondary metabolite synthesis. AT, acyltransferase for malonyl-CoA; AT_m_, acyltransferase for methylmalonyl-CoA; AT_e_, acyltransferase for ethylmalonyl-CoA; kr, inactive KR.

The chemical structure of nonthmicin suggested that their carbon skeletons are assembled from five malonyl-CoA, four methylmalonyl-CoA, and three ethylmalonyl-CoA molecules by a type-I PKS pathway. We therefore searched for a *t1pks* cluster consisting of 12 PKS modules. Among all of the four *t1pks* clusters present in *Actinomadura* sp. K4S16 (Table 5), *t1pks-4* cluster encoded six large PKSs and several enzymes related to secondary metabolite syntheses (Table 6, Fig 4a) and its assembly line contains 12 PKS modules. Substrates of AT domains in modules 1, 3 and 6 were predicted to be ethylmalonyl-CoA, whereas those in modules 5, 7, 8 and 9 were methymalonyl-CoA. According to the collinearity rule of type-I PKS pathways 17 and the chemical structure of nonthmicin, the polyketide backbone synthesized by the PKS assembly line was predicted as shown in Fig. 4b. The predicted structure is in good accordance with the nonthmicin backbone. The elongated polyketide chain is then converted to form three polyether moieties by an epoxidase and epoxide hydrase/cyclase(s) in a similar manner for the nanchangmycin biosynthesis 18. The tetronic acid part may be synthesized by ORFs K4S16_09_00680 to K4S16_09_00720 as proposed for tetronic acid-containing polyketides such as tetrocarcin A, chlorothricin, abyssomicin, and quatromicin 19-22, because these ORFs are orthologues of TcaDs, ChlM and ChlDs, AbyAs and QunDs. Two cytochrome P450s (K4S16_09_00590 and K4S16_09_00730) and a methyltransferase (K4S16_09_00740) are probably responsible for the introduction of one hydroxy group and one methoxy group to produce ecteinamycin. Chlorination to the tetronate moiety is presumably catalyzed by a halogenase (K4S16_09_00450) to yield nonthmicin. On the basis of these bioinfomatic evidences, we here propose the biosynthetic pathway of nonthmicin and ecteinamycin as shown Fig. 4b.

### Feeding experiments using labeled precursors

**Figure 5:**
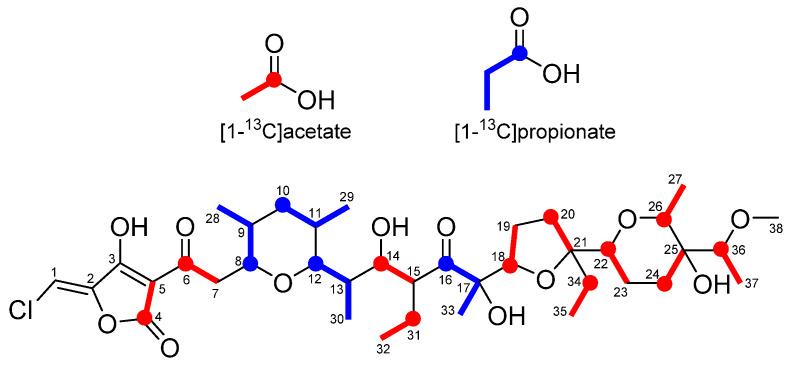
Incorporation of ^13^C-labeled precursors into nonthmicin.

To verify the predicted biosynthetic pathway for nonthmicin, feeding experiments were carried out using ^13^C-labeled precursors such as [1-^13^C]acetate and [1-^13^C]propionate. The signal intensities in ^13^C NMR spectrum of these labeled nonthmicin is shown in Table 7. Feeding of sodium [1-^13^C]acetate gave enrichments at twelve carbons at C4, C6, C14, C18, C20, C22, C24, C25, C26, C31, C34, and C36. [1-^13^C]propionate feeding enriched four methyl carbons at C28, C29, C30, and C33 (Fig. 5). These results unambiguously established that the polyether polyketide structure of nonthmicin is assembled from five malonyl-CoA, four methylmalonyl-CoA, and three ethylmalonyl-CoA. Labeling of C4 and C5 by acetate and non-labeling of C1, C2, and C3 by any precursors indicated that tetronic acid moiety is derived from one acetate and one glycerate units 23. These results also supported by annotated ORFs of *t1pks-4* cluster (K4S16_09_00690, K4S16_09_00700, K4S16_09_00710) (Fig. 4b, Table 6).

### Precursor-directed biosynthesis of bromo-analogue of nonthmicin

**Figure 6:**
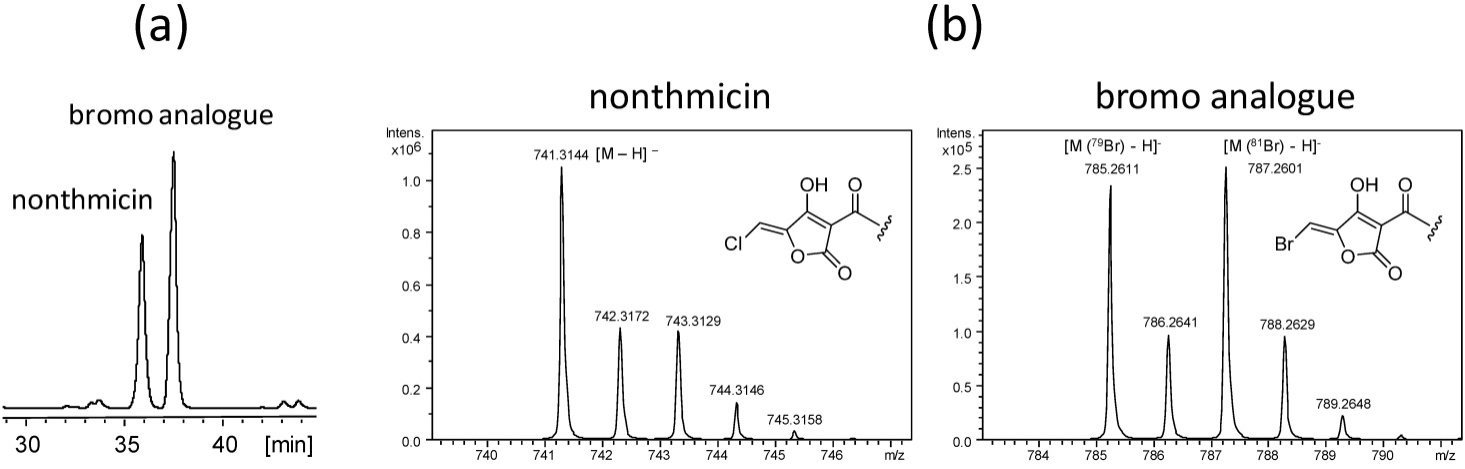
HPLC chromatogram of the bromine supplemented culture broth (a) and MS spectra of nonthmicin and bromine analogue (b).

A putative halogenase gene (K4S16_09_00450), showing 56% identity and 72% similarity of amino acid sequence to HalB from *Actinoplanes* sp. ATCC 33002, present in the nonthmicin biosynthetic gene cluster was expected to be responsible for the halogenation (Table 6). If this gene product is also active for bromine, it can be used for the precursor-directed biosynthesis of a brominated analogue. In fact, supplementation of sodium bromide into the culture resulted in the production of a new nonthmicin congener (Fig. 6a) in which the chlorine atom was replaced by the bromine atom. The structure of the bromo analogue was confirmed analysing data by MS (Fig. 6b) and NMR (data not shown).

## Conclusion

We successfully found the type-I PKS gene cluster for nonthmicin biosynthetic and proposed a plausible biosynthetic pathway by the genome analysis of *Actinomadura* sp. K4S16, a producer of nonthmicin. Incorporation experiments of ^13^C-labeled precursors also suggested that nonthmicin is biosynthesized by PKS pathway. These findings will provide significant information not only for the biosynthetic mechanism but also for the genetic engineering to synthesize more potential bioactive molecules based on the nonthmicin structure.

## Figures and Tables

**Table 1:** Project information

MIGS ID	Property	Term
MIGS 31	Finishing quality	Improved-high-quality draft
MIGS-28	Libraries used	454 shotgun library, Illumina paired-end library
MIGS 29	Sequencing platforms	454 GS FLX+, Illumina MiSeq
MIGS 31.2	Fold coverage	8.6 x, 73 x, respectively
MIGS 30	Assemblers	Newbler v2.8, GenoFinisher
MIGS 32	Gene calling method	Progidal
	Locus tag	K4S16
	GenBank ID	BDDE00000000
	GenBank date of release	Aug, 2019
	GOLD ID	Not registered
	BioProject	PRJDB4748
MIGS 13	Source material identifier	NBRC 110471
	Project relevance	Industrial

**Table 2:** Classification and general features of *Actinomadura* sp. K4S16 11

MIGS ID	Property	Term	Evidence code^a^
	Classification	Domain *Bacteria*	TAS 25
		Phylum *Actinobacteria*	TAS 26
		Class *Actinobacteria*	TAS 27
		Order *Actinomycetales*	TAS 27-30
		Suborder *Streptosporangineae*	TAS 27
		Family *Thermomonosporaceae*	TAS 27,30,31
		Genus *Actinomadura*	TAS 29,32
		Species undetermined (a new genomospecies)	This study
		strain: K4S16	TAS 4
	Gram stain	Not tested, likely positive	NAS
	Cell shape	Branched mycelia	IDA
	Motility	Not reported	
	Sporulation	Sporulating	IDA
	Temperature range	20 °C to 45 °C	IDA
	Optimum temperature	28 °C	IDA
	pH range; Optimum	5 to 8; 7	IDA
	Carbon source	Arabinose, fructose, glucose, mannitol, rhamnose, sucrose, xylose	IDA
MIGS-6	Habitat	Rice field soil	NAS
MIGS-6.3	Salinity	0 % to 2 % NaCl	IDA
MIGS-22	Oxygen requirement	Aerobic	IDA
MIGS-15	Biotic relationship	Free-living	IDA
MIGS-14	Pathogenicity	Not reported	
MIGS-4	Geographic location	Thailand	TAS 4
MIGS-5	Sample collection	March 13, 2010	NAS
MIGS-4.1	Latitude	Not reported	
MIGS-4.2	Longitude	Not reported	
MIGS-4.4	Altitude	Not reported	

a Evidence codes - IDA: Inferred from Direct Assay; TAS: Traceable Author Statement (i.e., a direct report exists in the literature); NAS: Non-traceable Author Statement (i.e., not directly observed for the living, isolated sample, but based on a generally accepted property for the species, or anecdotal evidence). These evidence codes are from the Gene Ontology project 33.

**Table 3:** Genome statistics of *Actinomadura* sp. K4S16

Attribute	Value	% of Total
Genome size (bp)	9,647,292	100
DNA coding (bp)	8,684,283	90.0
DNA G+C (bp)	6,982,736	72.4
DNA scaffolds	43	-
Total genes	9,068	100
Protein coding genes	9,004	99.3
RNA genes	64	0.7
Pseudogenes	-	-
Genes in internal clusters	4,198	46.6
Genes with function prediction	5,358	59.5
Genes assigned to COGs	7,701	85.5
Genes with Pfam domains	2,655	29.5
Genes with signal peptides	618	6.8
Genes with transmembrane helices	2,022	22.5
CRISPR repeats	1	-

**Table 4:** Number of genes associated with general COG functional categories

Code	Value	%age	Description
J	275	3.1	Translation, ribosomal structure and biogenesis
A	4	0.0	RNA processing and modification
K	1,158	12.9	Transcription
L	444	4.9	Replication, recombination and repair
B	3	0.0	Chromatin structure and dynamics
D	52	0.6	Cell cycle control, cell division, chromosome partitioning
V	202	2.2	Defense mechanisms
T	627	7.0	Signal transduction mechanisms
M	365	4.1	Cell wall/membrane biogenesis
N	46	0.5	Cell motility
U	87	1.0	Intracellular trafficking and secretion
O	232	2.6	Posttranslational modification, protein turnover, chaperones
C	540	6.0	Energy production and conversion
G	709	7.9	Carbohydrate transport and metabolism
E	851	9.5	Amino acid transport and metabolism
F	131	1.5	Nucleotide transport and metabolism
H	260	2.9	Coenzyme transport and metabolism
I	435	4.8	Lipid transport and metabolism
P	465	5.2	Inorganic ion transport and metabolism
Q	453	5.0	Secondary metabolites biosynthesis, transport and catabolism
R	1,459	16.2	General function prediction only
S	614	6.8	Function unknown
-	1,303	14.5	Not in COGs

The total is based on the total number of protein coding genes in the genome.

**Table 5:** PKSs and NRPSs encoded in each PKS or NRPS gene cluster of *Actinomadura* sp. K4S16

Cluster	ORF (K4S16_)	Domain	Predicted product
*t1pks-1*	13_05730	ACP-KS/AT/DH/KR/ACP/ACP-TE	phenolpthiocerol-like polyketide
*t1pks-2*	18_02330	KS/AT/KR	unpredictable
	18_02340	KS/AT	
*t1pks-3*	23_01060	KS/AT/KR/DH	enediyne-type polyketide
*t1pks-4*	09_00520	KS/AT/ACP-KS/AT/DH/KR/ACP-KS/AT/DH/ER/KR/ACP	nonthmicin, ecteinamycin
	09_00630	KS/AT/DH/KR/ACP-KS/AT/DH/ER/KR/ACP	
	09_00640	KS/AT/KR/ACP-KS/AT/KR/ACP	
	09_00660	KS/AT/ACP	
	09_00750*	KS/AT/KR/ACP-KS/AT/DH/ER/KR/ACP-KS/AT/DH/KR/ACP	
	09_00770*	KS/AT/KR/ACP	
*t2pks-1*	11_01150	KSα	aromatic polyketide
	11_01140	KSβ (CLF)	
	11_01130	ACP	
*t2pks-2*	09_01590	KSα	aromatic polyketide
	09_01600	KSβ (CLF)	
	09_01610	ACP	
*nrps-1*	11_07800	T-C/A_Cys_/T	peptide containing DHB, Cys, Gly, & mOrn
	11_07770	A_DHB_	
	11_07630	C/A_Gly_/T-C/A/T-C/A_Orn_/MT/T-C/T	
*nrps-2*	16_01040	T-C/A_Cys_/T-C/A_Cys_/T	St-Cys-Cys-mCys
	16_01050	C/A_Cys_/MT/T-TE	
*nrps-3*	24_00530	C/A_Ala_/T-C/A/T-C/T-C/A_Thr_/T/E	Ala-x-Thr-x-Thr-x
	24_00520	C/A/T-C/A_Thr_/T/E	
	24_00510	C/A/T-TE	

Substrates of A domains are shown by subscripts. DHB, dihydroxybenzoic acid; mOrn, methyl ornithine; St, starter molecule; mCys, methyl cysteine; x, unpredicted amino acid. *encoded in the complementary strand.

**Table 6:** ORFs of *t1pks-4* gene cluster responsible for the synthesis of nonthmicin

ORF^a^	Size (aa)	Deduced function	Closest protein homolog [origin]	Id/Si (%)^b^	Accession number
00450	551	halogenase	halogenase B [*Actinoplanes* sp. ATCC 33002]	56/72	AAQ04685
00460	188	flavin reductase	flavin reductase-like, FMN-binding [*Saccharopolyspora erythraea* NRRL 2338]	47/62	CAM04194
00470	220	two-component system response regulator	response regulator receiver protein, partial [*Microbispora rosea*]	65/78	WP_030509695
00480^c^	353	two-component system histidine kinase	hypothetical protein [*Herbidospora cretacea*]	43/63	WP_030450128
00490	906	transcriptional regulator	ATPase [*Microbispora* sp. ATCC PTA-5024]	41/54	ETK35445
00500^c^	576	3-hydroxybutyryl-CoA dehydrogenase	3-hydroxybutyryl-CoA dehydrogenase [*Streptomyces rapamycinicus* NRRL 5491]	56/65	AGP52928
00510^c^	340	3-oxoacyl-ACP synthase	3-oxoacyl-ACP synthase III [*Streptomyces* sp. C]	61/70	EFL20209
00520	4,859	polyketide synthase	polyketide synthase [*Streptomyces albus*]	54/64	AEZ53945
00530	442	crotonyl-CoA reductase	NADPH:quinone reductase [*Streptomyces albulus* PD-1]	74/83	EXU89989
00540	258	type-II thioesterase	thioesterase [*Streptomyces* sp. C]	59/69	EFL20221
00550	224	transcriptional regulator	hypothetical protein [*Actinomadura madurae*]	54/70	WP_021595170
00560	310	ABC transporter ATP-binding protein	hypothetical protein [*Lechevalieria aerocolonigenes*]	77/86	WP_030471487
00570	531	ABC transporter permease protein	hypothetical protein [*Actinopolymorpha alba*]	60/74	WP_020580181
00580	388	transcriptional regulator	LuxR-family transcriptional regulator [*Actinokineospora* sp. EG49]	42/52	EWC63761
00590	398	cytochrome P450	cytochrome P450 [*Streptomyces bingchenggensis* BCW-1]	52/68	ADI04501
00600^c^	136	epoxide hydrolase/cyclase	epoxide hydrolase [*Streptomyces longisporoflavus*]	53/67	ACR50776
00610	467	epoxidase	hypothetical protein SBI_01389 [*S. bingchenggensis* BCW-1]	58/70	ADI04510
00620^c^	183	epoxide hydrolase/cyclase	putative epoxide hydrolase/cyclase [*Streptomyces albus* subsp. *albus*]	53/66	CCD31907
00630	3,941	polyketide synthase	lasalocid modular polyketide synthase [*Streptomyces* sp. C]	54/64	EFL20211
00640	3,165	polyketide synthase	polyketide synthase [*Streptomyces graminofaciens*]	50/61	BAJ16467
00650	263	transcriptional regulator	putative pathway specific activator [*S. longisporoflavus*]	55/67	ACR50789
00660	1,563	polyketide synthase	Beta-ketoacyl synthase [*Streptomyces violaceusniger* Tu 4113]	50/62	AEM87323
00670^c^	576	ABC transporter permease protein	Putative exporter of polyketide antibiotics- like protein [*Thermomonospora curvata* DSM 43183]	45/60	ACZ00124
00680	342	3-oxoacyl-ACP synthase	3-oxoacyl-ACP synthase [*Streptomyces* sp. CNQ865]	65/77	WP_027767626
00690	637	glyceroyl-ACP biosynthesis protein	methoxymalonyl-ACP biosynthesis protein FkbH [*Streptomyces monomycini*]	60/69	WP_033040494
00700	75	ACP	ACP [*Amycolatopsis orientalis*]	72/81	AFI57025
00710	265	2-oxoglutarate dehydrogenase	acyltransferase [*Streptomyces olindensis*]	68/81	KDN76174
00720	365	hydrolase or acyltransferase	2-oxoacid dehydrogenase/acyltransferase [*Micromonospora chalcea*]	54/65	ACB37748
00730^c^	398	cytochrome P450	cytochrome P450 [*S. bingchenggensis* BCW-1]	53/71	ADI04501
00740^c^	288	methyltransferase	SAM-dependent methyltransferase [*Streptomyces* sp. NRRL F-2890]	48/59	WP_030734046
00750^c^	5,524	polyketide synthase	Acyl transferase [*S. violaceusniger* Tu 4113]	50/61	AEM84952
00770^c^	2,121	polyketide synthase	PieA2 [*Streptomyces piomogenus*]	51/62	AEZ54375

^a^ locus tag number after K4S16_09_; ^b^ identity/similarity; ^c^ encoded in the complementary strand.

**Table 7:** Incorporation of ^13^C-labeled precursors into nonthmicin

Position	δ_C_	Relative enrichments^a^	
		[1-^13^C]acetate	[1-^13^C]propionate
1	98.9	0.78	0.80
2	147.8	0.79	0.64
3	181.4	0.78	0.89
4	169.6	**2.56**	0.98
5	96.4	0.82	0.62
6	199.8	**2.28**	0.84
7	35.4	0.94	1.24
8	77.7	1.33	**4.25**
9	27.7	0.86	0.87
10	36.2	1.09	**4.85**
11	28.8	1.10	0.74
12	69.7	1.60	**4.70**
13	36.4	1.06	0.95
14	73.7	**2.25**	0.89
15	48.8	0.87	0.80
16	222.8	1.28	**4.49**
17	78.6	0.80	0.64
18	83.9	**2.41**	0.88
19	24.8	1.13	0.95
20	29.0	**2.14**	0.95
21	88.8	0.71	0.84
22	70.2	**2.64**	0.90
23	19.9	0.96	0.98
24	24.8	**2.19**	1.15
25	73.0	**2.49**	1.15
26	74.6	**2.47**	0.89
27	14.1	1.00	0.90
28	17.5	1.01	0.86
29	11.1	0.93	0.91
30	7.2	0.94	0.75
31	21.3	**3.11**	0.87
32	12.6	0.93	0.72
33	20.5	0.86	1.00
34	30.0	**2.19**	0.96
35	9.1	0.91	1.05
36	78.9	**3.25**	0.96
37	11.1	0.82	0.87
38	56.2	0.80	0.93

^a 13^C signal intensity of each peak in the labeled **1** divided by that of the corresponding signal in the unlabeled **1**, respectively, normalized to give an enrichment ratio of **1** for the unenriched peak of C27 and C33. The numbers in bold type indicate ^13^C-enriched atoms from ^13^C-labeled precursors.

## Abbreviations

A: adenylation
ABC: ATP-binding cassette
ACP: acyl carrier protein
Ala: alanine
AT: acyltransferase
ATP: adenosine triphosphate
BLAST: Basic Local Alignment Search Tool
C: condensation
CLF: chain length factor
CoA: coenzyme A
COG: Clusters of Orthologous Groups
Cys: cysteine
DH: dehydratase
DHB: dihydroxybenzoic acid
E: epimerase
ER: enoylreductase
Gly: glycine
ISP: International *Streptomyces* project
KS: ketosynthase
KR: ketoreductase
kr: inactive KR
MIGS: minimum information about a genome sequence
MT: methyltransferase
NBRC: Biological Resource Center, National Institute of Technology and Evaluation
NMR: nuclear magnetic resonance
NRPS: nonribosomal peptide synthetase
*nrps*: NRPS gene
PKS: polyketide synthase
*t1pks*: type-I PKS gene
*t2pks*: type-II PKS gene
T: thiolation
TE: thioesterase
Thr: threonine
